# Integrated Pest Management of Sclerotinia Stem Rot in Soybean: Current Strategies and Future Prospects

**DOI:** 10.3390/jof11120823

**Published:** 2025-11-21

**Authors:** Vivek Hemant Khambhati, Zhi-Yuan Chen

**Affiliations:** Department of Plant Pathology and Crop Physiology, Louisiana State University Agricultural Center, Baton Rouge, LA 70803, USA; vkhambhati@agcenter.lsu.edu

**Keywords:** RNA interference, chemical control, biological control, cultural practices, plant breeding

## Abstract

*Sclerotinia sclerotiorum* (Lib.) de Bary, the causal agent of Sclerotinia stem rot (SSR) or white mold, is a soil-borne hemibiotrophic fungus that causes substantial soybean yield losses worldwide. This pathogen infects over 400 plant species and persists in soil for extended periods through melanized sclerotia, which can survive under extreme environmental conditions. The wide host range, environmental adaptability, and longevity of sclerotia make SSR a persistent challenge in soybean production. No single management tactic provides reliable control, which underscores the importance of integrated pest management (IPM). Cultural practices such as crop rotation with non-hosts, optimized row spacing, adjusted seeding rates, and targeted irrigation are fundamental to reducing inoculum and modifying canopy microclimates to slow infection. Although genetic resistance remains partial, the deployment of cultivars with stable performance across environments contributes to disease suppression, particularly when combined with fungicide applications. However, fungicide efficacy is inconsistent and limited due to environmental concerns and potential resistance. Advances in disease modeling have improved the timing and precision of chemical control, while biological control agents and RNA interference approaches offer promising future options. This review synthesizes current IPM strategies for SSR and explores emerging alternatives to support sustainable soybean production.

## 1. Introduction to Sclerotinia Stem Rot

*Sclerotinia sclerotiorum* (Lib.) de Bary, the causal agent of Sclerotinia stem rot (SSR), is a soil-borne hemibiotrophic fungal pathogen that contributes to significant soybean (*Glycine max*) yield loss [1,2,3]. *S. sclerotiorum* is a member of the family Sclerotiniaceae, within the order Helotiales and the division Ascomycota. The fungus was first described in 1837 as *Peziza sclerotiorum* and has since undergone multiple taxonomic revisions before being assigned its currently accepted name in 1979 [1,3,4,5,6]. SSR, also known as white mold of soybean, is a widespread disease that infects all aboveground tissues of the soybean plant [3]. The disease can contribute to significant soybean yield losses due to reductions in seed number, seed weight, and seed quality, resulting in significant economic losses for growers [7,8]. Within the past ten years, SSR has consistently ranked among the top five diseases responsible for soybean yield losses in the northernmost states of the United States [9,10]. Between 1996 and 2023, estimated soybean yield losses caused by *S. sclerotiorum* totaled $5.5 billion without adjustment for inflation [11]. This review aims to describe the key biological characteristics of *S. sclerotiorum* that contribute to its persistence in soybean systems and to evaluate both conventional and emerging management strategies that can be integrated to offer a more effective SSR management program.

## 2. Signs and Symptoms

An effective IPM depends on routine scouting and accurate recordkeeping, with early detection of SSR signs and symptoms to serve as a primary defense against disease establishment and spread [12,13]. Apothecia are the first visible structures associated with *S. sclerotiorum* activity in the field and may be present before any foliar or stem symptoms are observed [12,14,15]. However, scouting for apothecia is extremely difficult [16], and these structures are sometimes misidentified as nonpathogenic fungi such as the bird’s nest fungus [12].

SSR symptoms generally begin to appear around the R5 growth stage and are first observed as white, cottony mycelium on the main stem and lateral branches [1,15]. This distinctive white growth, referred to as “white mold,” is a key diagnostic feature of *S. sclerotiorum* and is commonly observed across many hosts due to the pathogen’s broad host range [3,15]. Initial symptoms include water-soaked lesions with distinct margins that develop on stems, petioles, leaves, pods, and reproductive structures [1,3,17]. These lesions may rapidly expand above and below infected nodes. As infection progresses, affected tissues exhibit wilting, bleaching, and shredding [1,3,17]. The cottony hyphae frequently aggregate into compact masses that develop into black sclerotia, which may form either on the surface of infected tissue or internally within plant cavities such as the stem pith or inside pods [1,3,17]. Over time, infected stems become pale, fibrous, and stringy, and severe infection often results in wilting, lodging, and premature plant death [12,18].

Field distribution of *S. sclerotiorum* is typically patchy, and symptom expression is often delayed until late in the growing season, particularly in broad-acre production systems for grain and oilseed crops [19,20]. Despite variation in host response, the presence of white cottony mycelium and black sclerotia on aerial plant parts serves as a reliable diagnostic criterion for distinguishing SSR from other common soybean diseases [1,15,20].

## 3. Disease Cycle

The development of SSR in soybeans is driven by a combination of environmental, biological, and agronomic factors. The pathogen thrives under cool to moderate daily temperatures not exceeding 29 °C, and high moisture levels [21]. The pathogen persists in the environment in the form of hyphae, microconidia, ascospores, and most commonly as sclerotia. The biological function of microconidia remains unknown [22]. Ascospores are produced from apothecia, which are tan, cup-shaped sexual structures measuring approximately 3–6 mm in diameter [14], and are primarily dispersed within the field of origin, but may also be carried over long distances by wind [14,23,24]. The germination of ascospores and infection are favored by cool to moderate temperatures (15–25 °C), the presence of moisture on the leaf surface for at least two to four hours, and an exogenous nutrient source, typically provided by senescent or necrotic floral tissues [17,25].

In contrast, sclerotia are melanized, multicellular, seed-like structures that are resistant to extreme environmental conditions, including cycles of freezing and thawing, and can remain viable in the soil for five to eight years [17,26,27,28]. Sclerotia generally require a conditioning period of at least eight weeks at temperatures between 8 and 16 °C prior to germination [29,30]. Sclerotia can germinate through two distinct mechanisms, carpogenic or myceliogenic, depending on host species and environmental conditions [1]. Carpogenic germination results in the production of apothecia under shaded and moist topsoil conditions and subsequently ascospores to initiate aerial infections of aboveground plant parts [1,17,26,31]. Myceliogenic germination produces vegetative hyphae that can directly infect susceptible tissues, typically roots or crowns in certain crops [32,33].

**Figure 1:**
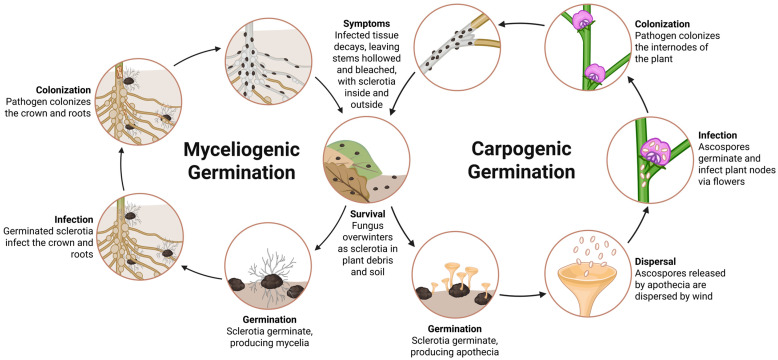
Disease cycle of *Sclerotinia sclerotiorum* in soybean. Overwintering sclerotia, which serve as primary inoculum, can undergo carpogenic or myceliogenic germination depending on environmental conditions. Carpogenic germination produces apothecia and airborne ascospores, which land on senescent flowers or injured tissue to initiate infection. Myceliogenic germination produces branched hyphae that directly infect crown or root tissue. As the disease progresses, colonized tissue will form dense mycelial growth and characteristic water-soaked lesions. New sclerotia develop within infected tissue, returning to the soil after plant decay to complete the cycle. The figure was created in BioRender (https://BioRender.com/j1d55xv), accessed on 14 November 2025, and adapted from Moellers [39].

In soybeans, the pathogen commonly infects during the flowering stages (R1 to R3), when senescent petals are present [15,34]. After landing on these tissues, ascospores germinate and initiate infection using both enzymatic degradation and mechanical penetration mechanisms, including the formation of compound appressoria and infection cushions [35,36,37]. Although stomatal entry and infection through wounds or contact with infected plants have been reported, the most common route of infection is through colonized, senescing flowers [15,17]. Following penetration, the fungus produces white to tan, hyaline, septate, branched, and multinucleate hyphae that colonize host tissues, leading to water-soaked lesions, wilting, tissue maceration, and plant death [17,38]. Sclerotia are subsequently produced within and on plant tissues, thus returning to the soil and completing the disease cycle [38]. Figure 1 illustrates and describes the disease cycle of *S. sclerotiorum* in soybean.

## 4. Host Range and Distribution

*S. sclerotiorum* is a globally important pathogen with a broad host range and widespread geographical distribution [40,41]. This hemibiotrophic fungus is among the most cosmopolitan and destructive plant pathogens, capable of infecting an estimated 425 documented plant species across 74 families, with a strong preference for dicotyledonous plants [2,42]. Major hosts include soybean, sunflower, oilseed rape (canola), edible dry bean, chickpea, peanut, lentil, and a wide variety of vegetable crops [3,42,43]. While traditionally considered a pathogen of dicotyledons, recent research has demonstrated its ability to colonize several monocotyledonous crops such as onion, tulip, rice, wheat, barley, oat, and maize, expanding its potential impact in diverse cropping systems [43,44].

The distribution of *S. sclerotiorum* extends across 95 countries on six continents, including Africa, Asia, Australia, Europe, North America, and South America [40]. The pathogen thrives in cool and moist environments, making temperate regions particularly susceptible to outbreaks of this disease [3]. In the United States, *S. sclerotiorum* has been reported in 44 states, with the Great Lakes Region frequently experiencing severe epidemics [40]. Its extensive host range and global presence make this pathogen a significant and enduring threat to the production of soybean and other susceptible crops. Understanding the impact of host range and geographical distribution on disease management strategies, such as crop rotations, etc., is essential for recommending effective management approaches, which are discussed in detail in subsequent sections of this review.

## 5. Management

**Figure 2:**
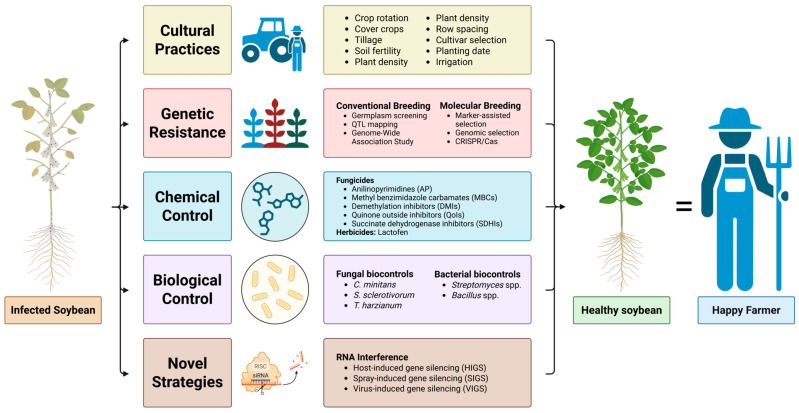
Graphic illustration of integrated pest management strategies to reduce *Sclerotinia sclerotiorum* infection in soybean. This figure was created using BioRender (https://BioRender.com/3defuky), accessed on 14 November 2025.

SSR management is difficult as no single management tactic provides complete control, making integrated strategies essential for effective management of the disease [12,34,45,46]. A robust IPM begins with cultural practices and deployment of genetically resistant cultivars to reduce initial inoculum, limit sclerotial germination, modify soybean canopy structures, and disrupt disease-favorable microclimates [12,34,47,48,49,50,51]. These cultural practices include rotation with non-host crops, optimized row spacing, adjusted seeding rates, and targeted irrigation during flowering [27,47,48,52,53,54,55]. Genetic resistance remains partial but is an important tool when selected based on multi-environment evaluations [46,56,57,58,59]. Chemical control, including fungicides and certain herbicides, should be reserved for periods of elevated disease risk and deployed in conjunction with other approaches to preserve their efficacy and minimize environmental impacts [34]. Biological control agents (BCAs) contribute another key component of SSR IPM by suppressing *S. sclerotiorum* infection and growth through mechanisms including nutrient competition, antagonistic metabolite production, direct reduction in sclerotial viability, and promotion of host defense responses via induced phytohormone pathways [45,60]. More recently, RNA interference-based strategies have gained attention as a novel, sustainable tool that can be integrated with conventional IPM tactics, providing precise gene-targeted suppression of *S. sclerotiorum* to enhance overall disease management outcomes [61]. When cultural, host-resistance, chemical, biological, and novel tactics are practiced synergistically and tailored to local environmental conditions and pathogen pressure, an effective, long-term, and sustainable management of SSR is achievable. Figure 2 illustrates the multi-layered holistic approach for the management of SSR in soybeans.

### 5.1. Crop Rotation, Cover Crops, and Tillage

Crop rotation as a sole management strategy is generally insufficient for controlling SSR due to the broad host range of *S. sclerotiorum* and the extended longevity of sclerotia in soil [26,27,28]. Nonetheless, forage legumes, such as alfalfa (*Medicago sativa*) and clovers, which exhibit lower susceptibility, and non-host crops, such as maize and small grains (including wheat, barley, oats, and sorghum), are suitable rotation options [12]. However, certain small grain crops can still be colonized by *S. sclerotiorum*, which may limit the effectiveness of crop rotation [12]. Therefore, when feasible, avoiding planting in infested or adjacent fields for four years or longer may provide greater reductions in inoculum pressure and reduce disease risk [43,44].

Cover crops are another potential tool for managing SSR. Small grain cover crops such as oat, wheat, and barley, when grown in association with soybean, have been shown to stimulate earlier emergence of apothecia compared with soybean grown alone, which may reduce the likelihood of infection by shifting apothecial development away from the most susceptible crop growth stages [12,62,63]. While certain cover crops may reduce disease incidence, others may serve as alternative hosts and increase inoculum levels [43]. In addition, cover crops influence soil moisture, nutrient availability, and shading of the primary crop, which can also affect SSR severity [64].

The influence of tillage practices on the development of SSR has been variable, with studies reporting fewer apothecia [27,52] and reduced disease severity in no-till systems despite a greater concentration of sclerotia near the soil surface [12,21,52,65], whereas other research has found no consistent relationship between tillage method and disease reduction [27,28]. It has been proposed that in no-till environments, sclerotia may be more susceptible to degradation compared with tilled soil due to increased exposure to ultraviolet radiation, desiccation, and predation [12]. Deeper tillage, such as moldboard plowing, can also temporarily reduce disease incidence by displacing sclerotia from the upper soil [28]. Given the variability in reported outcomes, additional research across different regions and soil types is needed to identify an optimal tillage strategy.

### 5.2. Cultural Practices to Modify Canopy Structures and Change Microclimates

#### 5.2.1. Managing Soil Fertility and Planting Time

Regular soil testing is recommended to avoid excessive nutrient applications in fields with a history of SSR, since high soil fertility, especially from nitrogen-rich fertilizers or manures, promotes vegetative growth and early canopy closure, which increases the chance for SSR development [12,50,51].

Only limited studies have addressed the influence of soybean planting date on the development of SSR [12,66]. It is widely recognized that early planting, use of late-maturing cultivars, and selection of genotypes with bushy growth habits or a tendency to lodge can promote rapid canopy closure and lead to extended periods of high humidity, which increase subsequent SSR development [34,59]. Nevertheless, the actual impact of planting date on disease incidence is inconsistent because SSR is strongly influenced by environmental conditions during soybean reproductive stages [21]. Studies in alfalfa have shown that earlier planting significantly reduced the severity of Sclerotinia crown and stem rot, as plants established before the emergence of apothecia were less susceptible to infection [67].

#### 5.2.2. Optimizing Row Spacing and Plant Density

Row spacing influences canopy microclimate and disease development [12]. Narrow rows close more quickly and increase the risk of SSR, whereas wider row spacing (≥51 cm) can delay canopy closure and reduce disease incidence in some situations [47,48]. Lower disease severity was observed at 76 cm row spacing compared to spacings between 25 and 38 cm, although this does not always correspond to higher yields [48].

Similarly, high plant densities, particularly those exceeding 432,100 plants per hectare (175,000 plants per acre), promote dense canopy formation, reduce airflow, and increase moisture retention [52,53]. These conditions facilitate apothecial germination and increase the severity of SSR [47,53]. Field studies have demonstrated that yields exceeding 95 percent of the maximum at 462,200 plants per hectare can be obtained at populations as low as 258,600 plants per hectare [52,53,68]. Therefore, population adjustments should be based on balancing yield potential with the risk of disease [12,34]. In other words, early planting, narrow row spacing, high plant populations, and high soil fertility accelerate canopy closure, creating an environment conducive to disease development [34].

#### 5.2.3. Cultivar Selection

Cultivar selection is a critical component of SSR management and should be guided by performance data collected across multiple locations and years to ensure consistent resistance under diverse environmental conditions [12,59]. Although no soybean cultivars possess complete resistance to *S. sclerotiorum*, several genotypes with partial resistance have been identified [56,58,59], and they often exhibit significantly lower disease incidence than susceptible cultivars, making them particularly valuable for fields with a history of SSR [12,56,58,59]. During elevated disease risk, growers are encouraged to prioritize cultivars with proven resistance in environments that closely match their local production conditions by engaging seed suppliers for information on resistant genetics.

Additionally, adoption of earlier maturity groups is recommended as it influences disease development [59]. Field studies conducted in Iowa revealed a linear relationship between maturity group and disease incidence, with later maturing cultivars exhibiting greater susceptibility [69]. Early maturing cultivars initiate flowering when the canopy remains relatively open, thereby decreasing the duration of favorable microclimatic conditions for fungal infection [56,70]. An upright plant architecture also contributes to reduced soil and canopy moisture, and thus less subsequent infection. These traits function as escape mechanisms that reduce pathogen establishment rather than conferring direct physiological resistance [59]. Ultimately, cultivar selection is most effective when incorporated within an IPM framework that simultaneously reduces inoculum levels and mitigates environmental conditions favorable for disease development [12].

#### 5.2.4. Irrigation Management

Irrigation can increase the incidence of SSR by providing the moisture required for sclerotial germination and subsequent infection [46]. Research has shown that restricting irrigation during flowering results in lower disease incidence compared with maintaining a biweekly irrigation schedule throughout the entire growing season [48]. Excessive irrigation beyond the levels required to sustain yield potential during flowering should be avoided to minimize moisture accumulation on the soil surface and within the crop canopy [48]. Maintaining low canopy moisture is essential for reducing the risk of SSR development. When irrigation is necessary, infrequent applications of greater volume are generally less conducive to disease than frequent, light watering [48]. Careful water management is particularly critical during the primary infection window, which extends from early flowering (R1) through early pod development (R3) [54]. Soybean plants typically require 0 to 7 days to progress from R1 to full flowering (R2) and 5 to 15 days to advance from R2 to R3, making this period the most important for minimizing excessive moisture to reduce SSR risk [54].

### 5.3. SSR Resistance Breeding

#### 5.3.1. Conventional Breeding Approaches for SSR Resistance

Breeding for genetic resistance to SSR has primarily relied on germplasm screening and quantitative trait loci (QTL) mapping [46]. More recently, genome-wide association studies (GWAS) have played a major role in identifying over 200 QTLs, with 14 validated for SSR resistance [71]. Despite these advances, breeding for SSR resistance remains challenging because resistance is a quantitatively inherited trait governed by multiple genes [8,72]. In addition, screening for resistance is complicated by environmental variability and differences in inoculum pressure, which can result in inconsistent infection levels across locations and seasons, underscoring the need for localized testing [46,57].

#### 5.3.2. Accelerating SSR Resistance with Molecular Breeding

Molecular breeding, also referred to as genomics-assisted breeding (GAB), has emerged as a powerful approach to enhance trait selection in soybean [73,74]. These techniques include marker-assisted selection (MAS) and genomic selection (GS) for QTL identification, as well as genome-editing technologies such as clustered regularly interspaced short palindromic repeats (CRISPR)/CRISPR-associated protein 9 (Cas9) for functional gene modification [73,75]. To date, MAS and GS have been used primarily to improve agronomic traits [76], with limited application to SSR resistance breeding. However, recent CRISPR/Cas9-based studies have demonstrated the potential for targeted improvement of SSR resistance. For example, overexpression of glutathione S-transferase (*GmGST*) enhanced soybean resistance to *S. sclerotiorum* [77], and functional analysis of the soybean 14-3-3 gene (*Glyma05g29080*) revealed its role in defense against infection [78]. These powerful tools remain underutilized in soybean SSR research and should be more widely integrated into modern breeding programs to accelerate the discovery and deployment of resistant traits.

### 5.4. Chemical Control

#### 5.4.1. Fungicide Options and Modes of Action for SSR Management

Multiple fungicide classes with distinct modes of action are available for SSR management. These include anilinopyrimidines (methionine biosynthesis inhibitors), methyl benzimidazole carbamates (MBCs) that disrupt microtubule formation and inhibit cell division, dicarboxamides which interfere with osmotic signal transduction, demethylation inhibitors (DMIs) that disrupt sterol biosynthesis and impair fungal cell wall development, quinone outside inhibitors (QoIs) that inhibit mitochondrial respiration, succinate dehydrogenase inhibitors (SDHIs) that block respiration by targeting succinate dehydrogenase, and electron transport uncouplers that disrupt oxidative phosphorylation, thereby impairing energy production [12,19,79,80,81,82]. MBC fungicides such as thiophanate methyl, SDHIs such as boscalid, DMIs including flutriafol, prothioconazole, and tetraconazole, and QoIs such as fluoxastrobin, picoxystrobin, and trifloxystrobin have been reported as effective options for SSR suppression [12,79,80,81,82]. Fungicide programs incorporating boscalid or picoxystrobin often provide the greatest disease suppression and yield benefits [83]. Fungicides that disrupt energy production tend to prevent spore germination, whereas those targeting cell division or structural components slow subsequent mycelial growth [12,34]. However, many of these chemistries have limited systemic movement and are therefore ineffective on untreated tissues, underscoring the importance of thorough spray coverage for better fungicide performance [12,34]. Flat fan spray nozzles producing fine to medium droplets (200 to 400 µm) have been shown to provide optimal canopy penetration and better protection of flowers from ascospore infection [12,84].

#### 5.4.2. Herbicide Use and Indirect SSR Suppression

Besides fungicides, the herbicide lactofen has also been evaluated as a supplemental chemical tool for SSR management [12,85]. Application of lactofen alters canopy architecture, potentially reducing humidity and modifying flowering patterns to create a less favorable environment for infection [86]. Additionally, lactofen can induce systemic acquired resistance in soybean, stimulating the production of antimicrobial phytoalexins such as glyceollin, which inhibit *S. sclerotiorum* growth [85,86,87,88,89]. Products containing lactofen (e.g., Cobra, Phoenix) are labeled to indicate potential SSR suppression. However, use of lactofen carries the risk of crop injury, including stunting and leaf malformation, and can reduce yield in seasons with low SSR pressure [81,85]. Phytotoxic effects reported in earlier studies included yield reductions of approximately 10% in the absence of disease [85]. Therefore, lactofen use should be carefully considered within an integrated program and timed appropriately to minimize negative crop impacts.

#### 5.4.3. Application Timing, Efficacy, and Resistance Management

Fungicides are predominantly preventive rather than curative, and their efficacy declines sharply if applied after symptom expression [12]. Therefore, application decisions must be made early, prior to visible disease development, to protect petals [12,19,34,90]. Spray programs are most effective when timed to coincide with the flowering window, particularly at the R1 growth stage (beginning bloom) [91]. Optimal timing varies with seasonal environmental conditions, and fungicide application is economically justified only when there is a moderate to high risk of infection [12,19,34,90]. In regions with high SSR pressure or crops with extended or indeterminate flowering periods, a second application may be warranted at later flowering stages to protect subsequent floral structures [92].

However, the effectiveness of fungicide sprays varies among fungicides, application timings, disease pressure, and environmental conditions, as documented in north central United States field studies [34,81,93,94], highlighting that fungicide application alone cannot achieve complete disease elimination and should be integrated with cultural and other management strategies [28,90,91]. In addition, the risk of resistance development in *S. sclerotiorum* populations must be considered since most fungicide classes used against SSR rely on a single mode of action [19]. Resistance of *S. sclerotiorum* to MBCs and dicarboxamides has been reported in some regions, and reduced sensitivity to SDHIs has been documented in France [19,95,96,97,98]. Strategic fungicide rotation with different modes of action, integration with cultural and biological controls, and careful application timing will remain critical to maintaining fungicide efficacy for SSR management [45].

### 5.5. Biological Control

#### 5.5.1. Fungal Control Agents

*Coniothyrium minitans* is the most extensively studied and commercially developed biological control organism for the management of *S. sclerotiorum* [60,99]. *C. minitans* is a specialized mycoparasite that attacks both hyphae and sclerotia of the pathogen [100]. *C. minitans* has lost the ability to infect plants and adapted to utilize sclerotia in the soil as its primary nutrient source [101]. Once sclerotia are colonized, they are degraded and rendered incapable of producing apothecia, thereby eliminating the production of ascospores that initiate disease epidemics [102]. Commercial formulations of *C. minitans* are available for agricultural use from various companies [99]. Application of *C. minitans* should be conducted at least three months before environmental conditions favor SSR development to allow sufficient colonization and degradation of sclerotia [103]. The product should be incorporated thoroughly into the soil to a depth of approximately 5 cm, and additional tillage that could expose uncolonized sclerotia should be avoided [45,103]. In various crops, including canola, sunflower, lettuce, cucumber, beans, and peanuts, field trials have reported reductions in disease symptoms ranging from 10 to 70% and reductions in sclerotial development of up to 95% [19,60,101,102,104,105,106,107]. In soybean, *C. minitans* has been reported to degrade up to 95% of sclerotia and reduce disease severity by approximately 68% [60]. Furthermore, Elsheshtawi et al. [108] found in greenhouse experiments that applications of a commercial formulation of *C. minitans* in combination with sublethal doses of a dicarboximide fungicide completely prevented symptom development in bean (*Phaseolus vulgaris* L.). These demonstrated efficacy, broad applicability, and compatibility with other control methods make *C. minitans* a valuable tool in the integrated management of *Sclerotinia* diseases.

Laboratory studies have consistently shown that various *Trichoderma* spp. interfere with the hyphal growth of *Sclerotinia* spp., parasitize sclerotia, and reduce the formation of apothecia [109]. Currently, there are more than 100 commercially registered *Trichoderma*-based biofungicides and plant growth–promoting products [110], and research into their biocontrol potential remains active. However, published evidence of field efficacy against *Sclerotinia* diseases is relatively limited [60]. Notable successes include reports of reductions in sclerotial density and symptom severity by up to 50–65% in cabbage and legumes [111,112,113]. In field and soil studies, the fungus *T. harzianum* reduced disease severity by 43% and 38%, and degraded sclerotia by up to 90% and 70%, respectively [113]. Commercial formulations of *T. harzianum* (PlantShield HC; BioWorks, Inc., Victor, NY, USA) also showed the potential for SSR management in limited field trials [113,114]. *S. sclerotivorum* is another fungal BCA that suppressed SSR in field trials [115]. Comparative trials have shown greater SSR disease severity reductions with *C. minitans* (68%) than with *Trichoderma harzianum* (35%) or bacterial biocontrol agent *Streptomyces lydicus* (43%) [113,116].

#### 5.5.2. Bacterial Control Agents

*Bacillus* spp. have been extensively investigated for their antagonistic activity against *Sclerotinia* spp., with numerous in vitro studies characterizing their mechanisms of suppression [117,118,119,120,121,122,123,124]. *B. subtilis* (BY2) and *B. megaterium* (A6) protected canola against disease caused by *S. sclerotiorum* in field trials, leading to significant yield increases [125,126]. Two foliar applications of *B. cereus* (SC1-1) reduced *S. sclerotiorum* incidence in canola by 71–80% [127]. In soybean, *B. subtilis* strains SB01 and SB24 reduced SSR severity under field conditions [128]. *B. amyloliquefaciens* and *B. cereus* reduced root rot symptoms in carnation by up to 88% when applied as root dips before transplanting [124], and several *Bacillus* spp. decreased disease severity in common beans and mustard in pot experiments [129]. Despite these encouraging findings, additional large-scale field evaluations are needed before widespread commercial recommendations can be made. Currently, several *B. amyloliquefaciens* and *B. subtilis* strains are marketed as biofungicides for use in leafy vegetables, root crops, legumes, and oilseeds for management of *Sclerotinia* spp. and other fungal pathogens [45].

*Streptomyces* spp. also exhibit considerable potential for *Sclerotinia* management due to their prolific production of antimicrobial secondary metabolites [130]. These bacteria often form endophytic or rhizosphere associations with plants, making them suitable candidates for agricultural biocontrol and plant growth promotion [131,132]. Commercial formulations of *S. lydicus* WYEC 108 have been registered as Actinovate^®^ AG (Novozymes BioAg Ltd., Saskatoon, SK, Canada) for soil applications to manage *S. sclerotiorum* and *S. minor* in brassicas, leafy vegetables, and legumes. In soybean field trials, soil drench applications of *S. lydicus* (Actinovate^®^ SP) reduced the disease severity by 30.8% and decreased sclerotia numbers in harvested plants by 93.8% compared with untreated controls [113].

#### 5.5.3. Challenges and Considerations for Biological Control Implementation

Biological control represents a promising component of integrated management programs for *S. sclerotiorum* in soybean production systems. Such integration reduces the reliance on chemical control, prolongs the efficacy of existing chemical modes of action, and mitigates the risk of selecting fungicide-resistant pathogen populations. However, biological control faces significant challenges related to the large-scale production and deployment of living organisms in agriculture [133]. BCAs tend to have a short shelf-life due to losing viability under non-optimum conditions. Recent advances in drying and packaging have significantly improved BCA longevity under low-moisture conditions [134,135]. Liquid formulations are typically preferred for their lower production costs and ease of use, although they have a shorter shelf-life [136]. Solid formulations offer greater stability and reduced transport costs, but are often more complex and costly to produce [136].

At present, BCAs are more expensive to produce than conventional chemical fungicides [137], largely due to the need for controlled environments, specialized equipment, trained personnel, and nutrient media for microbial growth [136]. Continued research may help reduce costs by identifying more potent BCA strains, improving production and formulation technologies, improving application methods, and increasing the stability of BCAs so they can persist in the environment through multiple growing seasons, thereby reducing the need for annual applications.

In addition, ecological risks need to be considered, as BCAs can disrupt existing microbial communities and ecological balances in crop systems [138]. The introduction of non-native microorganisms raises potential concerns regarding interactions with native species, resulting in greater regulatory scrutiny [136]. Consequently, BCAs must undergo rigorous evaluation and approval processes, particularly in the U.S. Environmental Protection Agency and the European Union, which increases development time and costs [139]. Continued investigation into the efficacy, consistency, and application strategies of fungal, bacterial, and other microbial agents is essential for future studies to fully integrate biological control into comprehensive management programs for SSR in soybean.

### 5.6. Disease Modeling

#### 5.6.1. Predictive Modeling and Decision Support Systems

The application of fungicides and BCAs is a costly component of soybean disease management, making it essential to apply them only when the risk of SSR is high. Predictive modeling has become a valuable tool to improve the timing of fungicide applications by identifying periods of elevated disease risk to reduce unnecessary applications and lower production costs. [83]. Epidemiological models have been developed for several *Sclerotinia* pathosystems, including canola, carrot, dry bean, Indian mustard, lettuce, and peanut, and similar approaches have been applied to soybean [140,141,142,143,144,145]. In soybeans, prediction efforts have focused on modeling apothecia development, as the presence of apothecia is strongly correlated with local disease incidence [24]. Two logistic regression models were created to account for contrasting microenvironments in irrigated and non-irrigated soybean production [13,41]. These models incorporate weather-based predictors such as 30-day moving averages of maximum air temperature and wind speed, with an additional row spacing variable included in the irrigated model [41]. The models demonstrated 67 to 70% accuracy in predicting apothecial presence during flowering, and subsequent refinements have further improved precision [41]. To enhance accessibility for producers, the models were integrated into a smartphone decision support system (DSS) called Sporecaster, which uses GPS coordinates and cloud-based weather data to provide field-specific risk assessments [146]. Based on multi-state validation studies, Sporecaster recommends fungicide applications when the probability of apothecia presence exceeds 10% in irrigated environments and 40% in non-irrigated fields [13]. This system achieved an accuracy of 87.9% for predicting SSR incidence at a 10% threshold in Wisconsin commercial fields, highlighting its reliability as a decision-making aid [13].

However, existing prediction models require further validation across diverse environmental and geographic contexts, as inconsistencies have been reported among published models. Moreover, current tools do not fully incorporate key environmental variables such as air temperature, relative humidity, soil temperature, precipitation, wind, dew point, and leaf wetness that may be necessary to develop a more robust forecasting capability for soybean systems [16]. In addition, agronomic factors including canopy structure, cultivar selection, planting density, row spacing, crop rotation, and tillage practices influence both carpogenic and myceliogenic germination of *S. sclerotiorum*, as well as disease severity and yield outcomes, and therefore should be integrated into future predictive frameworks [16].

#### 5.6.2. Adapting Models to Climate Change

The impacts of climate change add another layer of complexity to SSR management and IPM strategies. Rising temperatures, altered precipitation patterns, and increased frequency of extreme weather events are expected to directly and indirectly affect *S. sclerotiorum* biology, host physiology, and soil microbiome interactions [147,148]. These changes can shift the timing and intensity of apothecial development, modify disease-conducive microclimates within the canopy, and alter the geographic range of the pathogen [147]. Climate-driven stress on soybean plants may further increase susceptibility to infection, while altered soil conditions could influence the persistence and germination of sclerotia [147,149]. As a result, current IPM frameworks will need to be adapted to remain effective under changing climatic conditions. This includes refining disease prediction models, reassessing planting dates and cultivar selection, and developing climate-resilient strategies that integrate cultural, genetic, and biological controls with improved monitoring and forecasting systems.

### 5.7. RNA Interference as an Alternative Strategy for SSR Management

Current management practices, such as crop rotation, canopy management, biological control, and fungicide application, provide only partial or inconsistent suppression of diseases caused by *S. sclerotiorum*, highlighting the urgent need for innovative strategies that are more complete, consistent, and environmentally sustainable. RNA interference (RNAi) has emerged as a promising alternative in plant disease management by silencing essential genes in the pathogens involved in growth, development, and pathogenicity with high specificity. RNAi, also referred to as post-transcriptional gene silencing, is an evolutionarily conserved process that regulates target gene expression through sequence-specific degradation of target genes by small interfering RNAs (siRNA) of 21–24 nucleotides in length derived from double-stranded RNA (dsRNA) with the help of argonaute proteins and RNA-induced silencing complex (RISC) [150,151,152,153].

Presently, three distinct approaches are used to introduce dsRNAs into pathogens for disease suppression. Host-induced gene silencing (HIGS) is based on stable transformation of the plant genome to produce endogenous dsRNA molecules that target pathogen transcripts [154]. Spray-induced gene silencing (SIGS) is a non-transgenic alternative to HIGS, where exogenous dsRNA is directly applied to plant surfaces and subsequently taken up by plant pathogens either directly or indirectly [155]. Virus-induced gene silencing (VIGS), a transient alternative to HIGS, exploits engineered viral vectors or viral replicons to express dsRNA targeting pathogen genes and triggering RNAi responses in plants. Delivery can occur through agro-infiltration, DNA bombardment, or rub inoculation, resulting in replication of the viral genome and production of dsRNA sequences corresponding to the target gene [156].

#### 5.7.1. RNA Interference Strategies for SSR Control

The first report of HIGS-based approach to suppress *S. sclerotiorum* was published in 2015, when *Agrobacterium*-mediated transformation of *Nicotiana tabacum* introduced hairpin RNA targeting the fungal chitin synthase gene (*chs*), resulting in a 55.5% to 86.7% reduction in disease severity [157]. Since then, at least 27 fungal genes have been evaluated as potential targets through HIGS in multiple hosts including *Arabidopsis thaliana*, *Brassica napus*, *Nicotiana benthamiana*, and *N. tabacum* (Table S1) [157,158,159,160,161,162,163,164,165,166,167,168,169,170,171,172,173,174,175,176]. More recent studies have identified GDP-mannose pyrophosphorylase genes (*SsMPG1* and *SsMPG2*) as critical virulence factors, and transgenic *A. thaliana* and *N. benthamiana* expressing hairpin RNA against these genes displayed enhanced resistance, with *SsMPG2* RNAi lines showing markedly reduced lesion development [158]. Similarly, transgenic *A. thaliana* expressing hairpin RNA against a novel glycosylphosphatidylinositol-anchored protein (*SsGSP1*) that was shown to contribute to virulence, displayed significantly enhanced resistance with reduced lesion formation compared to controls [159]. Despite these advances, there are no reports to date of HIGS to protect soybean from *S. sclerotiorum*.

The first demonstration of SIGS against *S. sclerotiorum* was reported in 2018, in which 20 of 59 dsRNAs synthesized in vitro reduced lesion size of *S. sclerotiorum* on *B. napus* by 26 to 85%, and all 16 dsRNAs reduced lesion size on *A. thaliana* by 34 to 66% [177]. Since this initial report, a total of 87 genes from *S. sclerotiorum* have been evaluated as RNAi targets through SIGS (Table S2) [160,172,175,177,178,179,180,181,182,183,184]. More recent work has shown that topical application of dsRNAs targeting the transcription factor *SsPac1* and the MAP kinase *SsSmk1* suppressed disease development in *N. benthamiana* and *Brassica juncea*, with lesion reductions of up to 93% when both genes were silenced simultaneously [160]. Other targets such as the Cu/Zn superoxide dismutase *SsSod1*, chitin synthases *SsChs2* and *SsChs3*, and dicer-like proteins *SsDcl1* and *SsDcl2* were also evaluated in *N. benthamiana*, with decreases in lesion development and alterations in hyphal morphology and radial growth [160]. Collectively, these studies demonstrate that SIGS can effectively reduce *S. sclerotiorum* infection and that simultaneous silencing of multiple pathogenicity-related genes enhances control by disrupting fungal growth, development, and virulence.

Several studies have demonstrated that VIGS through the use of genetically modified plant viruses to transiently express dsRNA of *S. sclerotiorum* within the plant can also suppress pathogen growth or infection of hosts (Table S3) [161,185,186]. This strategy was first reported in soybean using a bean pod mottle virus vector modified to express oxaloacetate acetylhydrolase (*Ssoah1*), resulting in enhanced resistance to infection compared with control plants [185]. More recently, a viral RNAi vector was developed from tobacco rattle virus (TRV) to express double-stranded RNA targeting an ATP-binding cassette transporter gene (*SsBMR1*) [161]. In *N. benthamiana*, inoculation with the TRV2::BMR1 construct followed by *S. sclerotiorum* challenge significantly reduced lesion formation compared to plants treated with the TRV2::GFP control [161]. However, VIGS approaches have not achieved complete protection against disease caused by *S. sclerotiorum* in any host.

#### 5.7.2. Technical and Commercial Challenges in RNAi-Based Disease Management

The integration of RNAi-based technologies into the IPM framework varies depending on the approach. HIGS constructs can be incorporated into molecular breeding programs to develop soybean lines with enhanced resistance to SSR, while SIGS and VIGS approaches have the potential to serve as direct replacements for chemical control methods if they provide comparable levels of protection. Despite promising results, several challenges hinder the large-scale application of RNAi for soybean disease management. For HIGS, the development of homozygous transgenic plants requires several years, and the number of plant species that can be successfully transformed remains limited, especially for some crops [187]. In addition, regulatory restrictions and societal acceptance of genetically modified crops remain major hurdles [188]. Although SIGS is generally more acceptable, it has its own inherent limitations. The cost of dsRNA production was historically a major constraint but has declined substantially in recent years, from over $100 to less than $0.50 per gram [189,190]. Another key challenge is the poor stability of dsRNA under harsh environmental conditions, which has been addressed in recent studies through the use of nanomaterials that protect dsRNA and enhance its persistence [191]. Furthermore, efficient delivery and uptake of dsRNA by plants remain significant obstacles, although studies using adjuvants and liposomes have greatly enhanced foliar uptake and movement within plants [192,193]. All these need to be adequately addressed through formulation and optimization before cost-effective, large-scale field deployment becomes feasible [194].

While *S. sclerotiorum* is capable of internalizing dsRNA through endocytosis, the efficiency of uptake is inconsistent [195]. Nanoparticle-based encapsulation, including chitosan nanoparticles and layered double hydroxide nanosheets, offers potential solutions by protecting dsRNA molecules and enhancing uptake [196,197]. Environmental stability presents an additional challenge. DsRNA molecules degrade rapidly when exposed to ultraviolet radiation, rainfall, or nucleases, limiting their persistence under field conditions [196]. Encapsulation technologies, protective formulations, and microbial expression platforms such as engineered *Trichoderma* strains could provide extended stability [198].

Another concern is its off-target effects. Although RNAi is highly sequence-specific, siRNAs may inadvertently silence host transcripts or impact beneficial microbiota [199]. Bioinformatic prediction tools and extensive experimental testing are required to minimize these risks. Even though the likelihood of fungal pathogens developing resistance to foliar-applied dsRNA is extremely low and can be further minimized by combining dsRNAs targeting different genes, such instances have been reported in Colorado potato beetles (*Leptinotarsa decemlineata* Say) after exposure to high doses of dsRNA-coated leaves for nine generations [200]. Therefore, gene target selection is a critical component for successful SIGS application, as ideal candidates must be essential for pathogenicity, lack functional redundancy, and have minimal sequence similarity to genes from the host or beneficial microbes [177]. Advances in omics technologies have accelerated the identification of candidate targets, yet thorough validation is still required.

At present, no RNA-based fungicides have reached commercialization for crop protection against fungal pathogens. However, several companies, including GreenLight Biosciences, Trillium Ag, Innatrix, AgroSpheres, and Silvec, are actively pursuing the development of RNA-based pesticides. While most products currently focus on insect control, expansion into fungal disease management is underway. Integration of RNAi approaches with conventional management practices could provide a next-generation platform for sustainable disease control. Although technical, regulatory, and economic challenges remain, the demonstrated success of RNAi against *S. sclerotiorum* underscores its potential as a powerful tool for managing soybean white mold in an environmentally responsible manner.

## 6. Concluding Remarks and Future Directions

SSR remains a significant threat to soybean production and global food security. *S. sclerotiorum* is difficult to manage due to its complex biology and wide host range, necessitating an IPM approach for effective and sustainable control. Foundational strategies focus on preventing pathogen establishment through implementing cultural practices and planting more tolerant cultivars to reduce disease incidence [34]. However, additional field studies across diverse environmental conditions are required, as existing research often reports conflicting results. Continued progress in breeding is critical, given that only partially resistant cultivars are currently available. Breeding efforts can be accelerated through marker-assisted selection, and the latest CRISPR/Cas based genome editing technologies, genomic selection, and integrative multi-omics approaches [45,201]. Several fungicide classes are available for SSR management, but given the risk of fungicide resistance, chemical control alone is not a sustainable long-term solution [45]. BCAs have demonstrated strong potential under laboratory and greenhouse conditions, yet field-scale evidence in soybean remains limited, highlighting the need for expanded validation to support practical deployment [45,60]. Disease prediction models currently rely primarily on environmental variables, but greater accuracy may be achieved by incorporating available long-term datasets, varietal variations, and cultural practices, such as crop rotation, planting time, and plant density, to refine and validate predictive models across diverse production regions [16]. Advances in genomics and molecular biology are also enabling innovative control strategies, including RNA interference and targeted gene editing. Although RNAi-based fungicides are under active development, efficacy for SSR management has yet to be demonstrated under greenhouse or field conditions [61]. Future advances in artificial intelligence and machine learning can offer opportunities to develop image-based disease detection to further optimize the timing for applying various fungicides and biocontrol measures. Ultimately, effective SSR management will depend on multilayered strategies that integrate cultural practices, host resistance, biological and chemical tools, and emerging molecular and digital technologies.

## Data Availability

No new data were created or analyzed in this study. Data sharing is not applicable to this article.

## Supplementary Materials

The following supporting information can be downloaded at https://www.mdpi.com/article/10.3390/jof11120823/s1, Table S1: List of *Sclerotinia sclerotiorum* genes targeted with host-induced gene silencing (HIGS) [157,158,159,160,161,162,163,164,165,166,167,168,169,170,171,172,173,174,175,176]. Table S2: List of *Sclerotinia sclerotiorum* genes targeted with spray-induced gene silencing (SIGS) [160,172,175,177,178,179,180,181,182,183,184]. Table S3: List of *Sclerotinia sclerotiorum* genes targeted with virus-induced gene silencing (VIGS) [161,185,186].
