# Supplementary material for: Integrated Pest Management of Sclerotinia Stem Rot in Soybean: Current Strategies and Future Prospects
Source: J Fungi (Basel). 2025 Nov 21;11(12):823. doi: 10.3390/jof11120823 (PMC12734104; doi:10.3390/jof11120823)
Supplement: Supplementary file 1 [file jof-11-00823-s001.zip › Table S1.pdf]

Table S1. List of *Sclerotinia sclerotiorum* genes targeted with host-induced gene silencing (HIGS).

Gene Code	Gene Abbreviation	Protein Name/Function	Hosts	Reference(s)
SS1G_00134	*HSP70*	Heat shock 70 kDa protein	*N. benthamiana*	[162]
SS1G_00164	*MPG1*	GDP-mannose pyrophosphorylase	*A. thaliana*	[158]
SS1G_00966	*SOM1*	Transcription factor protein	*N. benthamiana*	[163]
SS1G_01703	*ABH*	Alpha/beta hydrolase 3; acetyl esterase/lipase	*B. napus*<br>*A. thaliana*	[164]<br>[165]
SS1G_02055	*HSF1*	Heat shock factor family protein; transcriptional factor	*N. benthamiana*	[162]
SS1G_02486	*CAF1*	EF-hand (EFh) domain-containing protein; Ca2+ binding protein	*A. thaliana*	[166]
SS1G_03194	*STE50*	Mitogen-activated protein kinase adaptor	*A. thaliana*<br>*N. benthamiana*	[167]<br>[167]
SS1G_03230	*GSP1*	Glycosylphosphatidylinositol-anchored protein	*A. thaliana*	[159]
SS1G_03860	*RAS2*	RAS-GTPases	*A. thaliana*<br>*N. benthamiana*	[168]<br>[168]
SS1G_04483	*BMR1*	ABC transporter G (ABCG) family protein	*A. thaliana*	[161]
SS1G_07355	*PAC1*	pH-responsive transcription factor	*A. thaliana*	[160]
SS1G_07569	*CAK1*	Protein kinase family protein	*N. benthamiana*	[169]
SS1G_07873	*NOB1*	20S-pre-rRNA D-site endonuclease	*A. thaliana*	[170]
SS1G_08218	*OAH1*	Oxaloacetate acetylhydrolase	*A. thaliana*<br>*B. napus*<br>*B. napus*	[171]<br>[172]<br>[173]
SS1G_08263	*MSB2*	Beta strand repeat-containing protein; membrane protein	*N. benthamiana*	[163]

SS1G_08534	*TRX1*	Thioredoxin	*A. thaliana*<br>*N. benthamiana*	[174]<br>[174]
SS1G_09020	*CBH*	Cellobiohydrolase	*B. napus*	[172]
SS1G_10167	*PG1*	Endo-polygalacturonase	*A. thaliana*<br>*B. napus*	[166]<br>[172]
SS1G_11299	*GAP1*	RAS-GTPase activating protein	*A. thaliana*<br>*N. benthamiana*	[168]<br>[168]
SS1G_11866	*SMK1*	Mitogen-activated protein kinase	*A. thaliana*<br>*N. benthamiana*	[160]<br>[163]
SS1G_12062	*MNO1*	FAD-dependent monooxygenase	*A. thaliana*<br>*B. napus*	[175]<br>[175]
SS1G_12594	*PDE2*	3'5'-cyclic nucleotide phosphodiesterase; cAMP phosphodiesterase	*N. benthamiana*	[176]
SS1G_13453	*MPG2*	GDP-mannose pyrophosphorylase	*A. thaliana*	[158]
SS1G_13677	*SNF5*	Subunit of the SWI/SNF complex	*N. benthamiana*	[162]
SS1G_13801	*RAS1*	RAS-GTPases	*A. thaliana*<br>*N. benthamiana*	[168]<br>[168]
SS1G_14116	*CHS*	Chitin synthase	*N. tabacum*	[157]
SS1G_14133	*ITL*	Integrin protein	*A. thaliana*	[166]
