# Supplementary material for: Integrated Pest Management of Sclerotinia Stem Rot in Soybean: Current Strategies and Future Prospects
Source: J Fungi (Basel). 2025 Nov 21;11(12):823. doi: 10.3390/jof11120823 (PMC12734104; doi:10.3390/jof11120823)
Supplement: Supplementary file 1 [file jof-11-00823-s001.zip › Table S2.pdf]

Table S2. List of *Sclerotinia sclerotiorum* genes targeted with spray-induced gene silencing (SIGS).

Gene Code	Gene Abbreviation	Protein Name/Function	Hosts	Reference(s)
SS1G_00334	*AGO2*	Argonaute 2	*B. napus*<br>*N. benthamiana*	[178]<br>[179]
SS1G_00363	-	Poly(A)-binding domain-containing protein	*B. napus*	[177]
SS1G_00435	-	Imidazoleglycerol-phosphate hydratase	*B. napus*	[177]
SS1G_00509	-	Glycosyl hydrolase family 10 protein	*B. napus*	[177]
SS1G_00699	*SOD1*	Cu/Zn superoxide dismutase	*N. benthamiana*	[160]
SS1G_01348	-	Orotate phosphoribosyltransferase	*B. napus*	[177]
SS1G_01605	-	Proteasome regulatory subunit rpn-8	*B. napus*	[177]
SS1G_01703	*ABH*	Alpha/beta hydrolase 3; acetyl esterase/lipase	*A. thaliana*<br>*B. napus*	[177]<br>[177,180]
SS1G_02468	-	DEAD/DEAH box-containing ATP-dependent helicase	*B. napus*	[177]
SS1G_02495	-	Peroxidase	*A. thaliana*<br>*B. napus*	[177]<br>[177]
SS1G_02791	-	Zn(II)2Cys6 transcription factor domain-containing protein	*B. napus*	[177]
SS1G_03208	-	PRP8 family protein; 35S U5 snRNP spliceosome subunit	*A. thaliana*	[177]
SS1G_03270	*SNF7*	Snf7 family protein; vacuolar protein sorting protein	*B. napus*	[177]
SS1G_03348	-	Mov34/MPN/PAD-1 family protein containing COP9 signalosome subunit	*B. napus*	[177]
SS1G_03852	-	PH (Pleckstrin homology) domain-containing protein	*B. napus*	[177]

SS1G_03857	*CHS2*	Chitin synthase	*N. benthamiana*	[160]
SS1G_03991	-	Transcription cofactor	*A. thaliana*	[177]
SS1G_03992	*MED12*	Transcription mediator complex subunit	*B. napus*	[177]
SS1G_04001	-	Cytochrome c oxidase subunit Via	*B. napus*	[177]
SS1G_04040	*NMT1*	N-myristoyl transferase	*B. napus*	[177]
SS1G_04144	*DCTN1*	Dynactin	*B. oleracea* var. *acephala*<br>*L. sativa* var. *ramosa*<br>*B. napus*	[181]<br>[181]<br>[182]
SS1G_04479	-	Nitrite reductase containing Rieske domain	*B. napus*	[177]
SS1G_04532	-	WD40 repeat domain-containing protein	*B. napus*	[177]
SS1G_04551	-	Pectin methylesterase	*B. napus*	[177]
SS1G_04805	*CYP51*	Sterol 14α-demethylase	*B. napus*<br>*B. juncea*	[177]<br>[183]
SS1G_04953	-	Sec1 family protein; vesicle trafficking	*B. napus*	[177]
SS1G_04966	-	RNA polymerase-associated RTF1 family protein; transcription factor	*A. thaliana*	[177]
SS1G_05366	*MOB1*	Mob1/phocein family protein	*B. napus*	[177]
SS1G_05491	*CYP58*	Cytochrome P450 family 58-like fungal cytochrome P450s	*B. napus*	[180]
SS1G_05583	-	Malate synthase	*B. napus*	[177]
SS1G_05899	*TRR1*	Thioredoxin reductase	*A. thaliana*<br>*B. napus*	[177]<br>[177]
SS1G_05979	-	Coenzyme Q-binding protein	*B. napus*	[177]

SS1G_06055	-	4-Hydroxybenzoate polyprenyltransferase	*B. napus*	[177]
SS1G_06305	-	Histidine kinase	*B. napus*	[177]
SS1G_06421	*RIO1*	Serine/threonine protein kinase	*B. napus*	[177]
SS1G_06487	*TIM44*	Mitochondrial import inner membrane, translocase subunit	*A. thaliana*<br>*B. napus*	[177]<br>[177]
SS1G_06830	-	Amino acid permease (GABA permease)	*A. thaliana*	[177]
SS1G_07355	*PAC1*	pH-responsive transcription factor	*B. juncea*<br>*N. benthamiana*	[160]<br>[160]
SS1G_07456	-	DUF2841 domain-containing protein; Sec24-related protein	*B. napus*	[177]
SS1G_07715	*SAC1*	Suppressor of actin	*B. oleracea* var. *acephala*<br>*L. sativa* var. *ramosa*<br>*B. napus*	[181]<br>[181]<br>[182]
SS1G_07873	*NOB1*	20S-pre-rRNA D-site endonuclease	*A. thaliana*<br>*B. napus*	[177]<br>[177]
SS1G_07958	-	Major facilitator superfamily (MFS) transporter	*B. napus*	[177]
SS1G_08020	-	Chitinase, GH18 family	*B. napus*	[177]
SS1G_08218	*OAH1*	Oxaloacetate acetylhydrolase	*B. napus*<br>*B. juncea*	[172,177,180]<br>[183]
SS1G_08265	*CHS3*	Chitin synthase	*N. benthamiana*	[160]
SS1G_08431	-	Cytidylyltransferase family protein; CDP-diacylglycerol synthase	*B. napus*	[177]
SS1G_09020	*CBH*	Cellobiohydrolase	*B. napus*	[172]
SS1G_09028	*VPS51*	Vacuolar protein sorting 51	*B. oleracea* var. *acephala*<br>*L. sativa* var. *ramosa*<br>*B. napus*	[181]<br>[181]<br>[182]

SS1G_09088	*TOM40*	Mitochondrial import receptor subunit TOM40 family protein	*B. napus*	[177]
SS1G_09261	-	Kinetochore Spc7 family protein	*B. napus*	[177]
SS1G_09394	-	Bifunctional phosphopantothenoylcysteine synthetase/decarboxylase (CoaBC) family protein	*B. napus*	[177]
SS1G_09665	-	Inositol-pentakisphosphate 2-kinase	*B. napus*	[177]
SS1G_09680	*NOC3*	Nucleolar complex-associated protein 3	*A. thaliana*	[177]
SS1G_09897	*CDC25*	Mitotic inducer, protein phosphatase; Cdc25	*B. napus*	[177]
SS1G_09997	*BGT1*	Glycosyltransferase	*B. napus*	[180]
SS1G_10049	*TIM17*	Mitochondrial import inner membrane translocase, subunit TIM17	*B. napus*	[177]
SS1G_10108	-	Glutathione S-transferase	*B. napus*	[177]
SS1G_10167	*PG1*	Endo-polygalacturonase	*B. napus*	[172,177]
SS1G_10369	*DCL2*	Dicer-like protein	*B. oleracea* var. *acephala*<br>*L. sativa* var. *ramosa*<br>*N. benthamiana*	[181]<br>[181]<br>[160]
SS1G_10396	-	CsbD (Bacterial stress response), mismatch base repair	*B. napus*	[177]
SS1G_10456	-	SUR7/PalI family protein	*B. napus*	[177]
SS1G_10698	-	Glycoside hydrolase family 28 protein	*B. napus*	[177]
SS1G_11049	-	Glycosylphosphatidylinositol (GPI) transamidase	*B. napus*	[177]

SS1G_11468	*ERP1*	Ethylene pathway repressor protein 1	*N. benthamiana*	[184]
SS1G_11704	-	LUC7 domain-containing protein; U1 snRNP component	*B. napus*	[177]
SS1G_11723	*AGO4*	Argonaute 4	*B. napus*	[178]
SS1G_11780	-	Amino acid permease (GABA permease)	*B. napus*	[177]
SS1G_11835	-	Ubiquinol-cytochrome c reductase complex assembly factor 2 (UQCC2)	*B. napus*	[177]
SS1G_11866	*SMK1*	Mitogen-activated protein kinase	*B. juncea*<br>*B. napus*<br>*N. benthamiana*	[160]<br>[180]<br>[160]
SS1G_11912	*NEP2*	Necrosis/ethylene inducing peptide 2	*A. thaliana*<br>*B. napus*	[177]<br>[177]
SS1G_12021	-	1,3-beta-glucan synthase	*A. thaliana*	[177]
SS1G_12062	*MNO1*	FAD-dependent monooxygenase	*A. thaliana*<br>*B. napus*<br>*N. benthamiana*	[175]<br>[175]<br>[175]
SS1G_12078	-	Slx1-Slx4 complex	*B. napus*	[177]
SS1G_12152	-	Acetyl-CoA acetyltransferase	*B. napus*	[177]
SS1G_12640	-	Thioredoxin domain-containing protein	*A. thaliana*	[177]
SS1G_12992	-	Transglutaminase family protein	*A. thaliana*	[177]
SS1G_13314	-	Nuclear transport factor 2 (NTF2) family protein	*B. napus*	[177]
SS1G_13339	-	Choline/carnitine o-acyltransferase	*B. napus*	[177]
SS1G_13392	-	N-acetylneuraminic acid mutarotase	*B. napus*	[177]

SS1G_13702	*TIM23*	Mitochondrial import inner membrane translocase, subunit TIM23	*A. thaliana*	[177]
SS1G_13720	-	Sugar porter family major facilitator superfamily (MFS) transporter protein	*B. napus*	[177]
SS1G_13746	-	Pex2/pex10/pex12 family protein	*A. thaliana*	[177]
SS1G_13747	*DCL1*	Dicer-like protein	*B. oleracea* var. *acephala*<br>*L. sativa* var. *ramosa*<br>*N. benthamiana*	[181]<br>[181]<br>[160]
SS1G_13982	-	Alpha/beta hydrolase	*B. napus*	[177]
SS1G_14184	*CBD*	Chitin-binding domain protein	*B. napus*	[180]
SS1G_14257	-	20S proteasome beta type 7 subunit/N-terminal nucleophile hydrolase	*B. napus*	[177]
SS1G_14298	-	Basic Helix-Loop-Helix-zipper transcription factor	*B. napus*	[177]
