# Supplementary material for: Integrated Pest Management of Sclerotinia Stem Rot in Soybean: Current Strategies and Future Prospects
Source: J Fungi (Basel). 2025 Nov 21;11(12):823. doi: 10.3390/jof11120823 (PMC12734104; doi:10.3390/jof11120823)
Supplement: Supplementary file 1 [file jof-11-00823-s001.zip › Table S3.pdf]

Table S3. List of *Sclerotinia sclerotiorum* genes targeted with virus-induced gene silencing (VIGS).

Gene Code	Gene Abbreviation	Protein Name/Function	Hosts	Reference(s)
SS1G_04483	*BMR1*	ABC transporter G (ABCG) family protein	*N. benthamiana*	[161]
SS1G_08218	*OAH1*	Oxaloacetate acetylhydrolase	*G. max*	[185]
SS1G_11468	*CND1*	Magnaporthe appressoria-specific homolog gene	*N. benthamiana*	[186]
